# The social licence for research: why *care.data* ran into trouble

**DOI:** 10.1136/medethics-2014-102374

**Published:** 2015-01-23

**Authors:** Pam Carter, Graeme T Laurie, Mary Dixon-Woods

**Affiliations:** 1 Health Sciences, University of Leicester, Leicester, UK; 2 Department of Law, University of Edinburgh, Edinburgh, UK

**Keywords:** Research Ethics, Informed Consent, Confidentiality/Privacy, Information Technology, Regulation

## Abstract

In this article we draw on the concept of a social licence to explain public concern at the introduction of *care.data*, a recent English initiative designed to extract data from primary care medical records for commissioning and other purposes, including research. The concept of a social licence describes how the expectations of society regarding some activities may go beyond compliance with the requirements of formal regulation; those who do not fulfil the conditions for the social licence (even if formally compliant) may experience ongoing challenge and contestation. Previous work suggests that people's cooperation with specific research studies depends on their perceptions that their participation is voluntary and is governed by values of reciprocity, non-exploitation and service of the public good. When these conditions are not seen to obtain, threats to the social licence for research may emerge. We propose that *care.data* failed to adequately secure a social licence because of: (i) defects in the warrants of trust provided for *care.data*, (ii) the implied rupture in the traditional role, expectations and duties of general practitioners, and (iii) uncertainty about the status of *care.data* as a public good. The concept of a social licence may be useful in explaining the specifics of *care.data*, and also in reinforcing the more general lesson for policy-makers that legal authority does not necessarily command social legitimacy.

## Background

Encouraging more and better health-related research is now firmly established as a policy goal in England.^1–3^ A crucial feature of the current policy drive is an emphasis on the use of the distinctive infrastructure of the personal medical records of individuals registered with the National Health Service (NHS). Researchers have long argued that, contrary to the public interest, these records have remained under-exploited as a research resource.^4^ Yet the recent launch of *care.data—*an initiative to extract data from NHS primary care medical records in England unless patients have purposefully opted out, in part to facilitate research—has proved deeply controversial. Within weeks of the start of a public information campaign run by NHS England, public and professional concern had become so overwhelming that in February 2014 the implementation of the scheme was suspended for 6 months.^5^ Our aim in this article is not to assess the rights and wrongs of *care.data—*others have already offered such critiques^6^—nor to offer an in-depth analysis of the legal background, but rather to show how the concept of a social licence^7^ can help to explain the challenges faced by *care.data*.

Although *care.data* has numerous aims (box 1), we focus specifically on its research purposes. We begin by offering some brief background on the use and regulation of routine medical data before introducing the concept of a social licence.

Box 1 **Aims of *care.data***

NHS England has described the *care.data* service as: ‘…a new, modern data system for the NHS in England. Known as *care.data*, its purpose will be to provide timely, accurate information to citizens, clinicians and commissioners about the treatments and care provided by the NHS’ (http://www.england.nhs.uk/wp-content/uploads/2013/05/ces-tech-spec-gp-extract.pdf).

The aims of the *care.data* programme are sixfold:

1. To support patients’ choice
2. To advance customer services
3. To promote greater transparency
4. To improve outcomes
5. To increase accountability
6. To drive economic growth by making England the default location for world-class health services research.

NHS, National Health Service.

## The use and regulation of medical records for research

Researchers have long relied on access to personal medical information routinely collected during the course of patient care in order to conduct studies, including clinical trials and epidemiological research. However, the repurposing of routinely collected data for research is not without risk to relevant values,^8^ and measures such as anonymisation (even when possible) do not solve all ethical, legal and technical problems; people may, for example, have religious or moral objections to particular studies^5^ or concerns about stigma and breaches of privacy.

Accordingly, researchers’ access to, and use of, personal data has traditionally been subject both to tight controls and to efforts to promote public and patient confidence in those controls. The Data Protection Act 1998, which implements the European Data Protection Directive, has always recognised ‘medical purposes’ as a legal basis for processing personal data from medical records, subject to a proportionality test. While it contains a ‘research exemption’, it does not absolve data controllers completely of their obligations under the law.^9^ ^10^ Data protection has been bolstered by the requirement, introduced in 1999, that each NHS organisation have a designated senior individual (known as a Caldicott Guardian) with responsibility both for the confidentiality of patient information—which is protected by the common law—and for enabling appropriate information sharing. These legal measures also operate alongside guidance and codes of practice issued and updated regularly by the professional regulators, by the NHS Research Governance Framework,^11^ by a system of research ethics committees, and by a variety of other mechanisms.

Nonetheless, access to and secondary use of medical information for research purposes has continued to be a fraught and contested activity, prone to concern over its ethical standing.^12^ The tensions were discussed extensively in a 2006 report of the Academy of Medical Sciences (AMS),^4^ in a document that can be seen as an attempt at advocacy on the part of a clinical research community that saw itself as beleaguered and misunderstood. The report offered multiple examples of the benefits of research that uses routinely collected personal data, including its possibilities as a means of answering primary research questions, as a prelude to clinical trials, as a means for disease surveillance, and as a resource for identifying potential participants in trials or other studies. But such activities were, the AMS report argued, continually thwarted by the complexities and frustrations of multiple legal, regulatory and professional restrictions.

The AMS report^13^ and others, while accepting the need for regulation, argued that the regulatory environment for the use of personal data for health research in England was, by 2006, not in the public interest: it was disproportionate relative to the risks and benefits involved and involved multiple overlapping layers, institutions and actors as well as contested and sometimes conflicting standards and expectations for research governance. While accepting the broad principle of the need for regulation, the report suggested that NHS organisations were overly cautious about legal risk in areas of interpretive uncertainty, and tended to default to a conservative approach. The way the rules relating to data protection were understood, interpreted and applied by NHS organisations, research ethics committees and others was a focus of particular criticism. The overall effect, the report and others argued, was that NHS organisations were generally reluctant to share personal information without explicit consent from patients or anonymisation of data to remove them from the reach of data protection legislation: the dichotomous ‘consent or anonymise’ approach was the norm.

Yet this was not the only possible approach, nor was it required by law. Under what was Section 60 of the Health and Social Care Act (2001) (later Section 251 of the NHS Act 2006), a Patient Information Advisory Group (PIAG), acting on behalf of the Secretary of State, was allowed to approve processing of patient information without seeking patients’ consent for research purposes. However, the AMS report criticised the PIAG's approach, processes and membership, and its alleged tendency to stress its role in protecting privacy or confidentiality ‘without equal emphasis on the benefits derived from well-conducted research’.^4^ PIAG was argued to suffer from mission creep, applying a stricter standard of absolute and proven necessity rather than assessing whether applications involved a proportionate and justifiable interference in privacy.

Central to the AMS report's argument for reform was that PIAG appeared to adopt a more conservative approach than patients and the public would favour. It claimed strong public approval and support for research using medical records, on the grounds that the risks involved were low in comparison with the likely benefits and in comparison with other forms of research (such as clinical trials). This claim was in keeping with the influential (although not necessarily uncontested) view that over-emphasis on individual consent fails to balance a broader range of ethical principles and the argument that people have a responsibility to participate in research.^14^ ^15^ The tenor of the arguments was that the proper solution to the challenges surrounding use of routine medical data for research purposes might then be one of adjusting the regulatory environment to accommodate what the public would support.^16^

## A social licence for research involving medical records?

Based on the reasoning that the public would accept and endorse a less restrictive approach to the use of medical records for research purposes than the custodians of the system realised, the AMS's clarion call was to ‘be bold’. Although it did not use the term explicitly, the core of the AMS argument was that the *social licence* for the use of medical records for research was more permissive than the operation of the regulatory environment allowed.

The concept of a social licence is discussed in two distinct literatures. One important literature derives from the work of the sociologist Everett Hughes^17^ who describes the relationship between a profession and society in terms of the two concepts of licence and mandate. For Hughes, licence is granted to certain occupational groups to carry out particular activities; a mandate is claimed by members of the group to define for itself proper conduct in relation to matters concerned with their work.^7^ A second, and mostly distinct, corpus of work on corporate social responsibility describes the concept of the ‘social licence to operate’ as the expectations of society regarding the conduct and activities of corporations that go beyond the requirements of formal regulation.^18^ Thus, industries with significant environmental impact may find that operating within the law but outside the boundaries of social approval can result in corporate damage—for example, by having a negative impact on a company's brand or provoking new and restrictive regulation. The Australian mining industry is often offered as an example of an industry required to earn a social licence, and maintain it, by behaving in a trustworthy and responsible way; if it were otherwise, it would face ongoing challenge and costly delays and interference in its activities.^19^

Some analogies have been drawn between a social licence for the mining of minerals and for the process of data mining.^20^ Regarding these and similar activities, what the social licence emphasises is the possible need for those (whether they are public or private bodies, or specific occupational groups) undertaking activities likely to provoke public disquiet to go ‘beyond compliance’ with legal requirements. We propose that a poorly informed understanding of the social licence for secondary use of personal medical data, and a failure to recognise that legal authority might not be enough to secure the social licence, seems to have been at the heart of the controversy underlying *care.data*.

### Legal authority for *care.data*

The Health and Social Care Act 2012 established the Health and Social Care Information Centre (HSCIC) as an Executive Non-Departmental Public Body empowered to obtain patient-identifiable information from general practices. Practices themselves were, under their NHS contract, obliged to transfer the data to HSCIC unless patients explicitly opted out. Using a centrally managed General Practice Extraction Service (GPES) based on commercially supplied software, *care.data* was intended to gather data monthly, capturing most routine general practice consultations. The data would then be made available in aggregate form to the HSCIC, with six aims (box 1) identified for their use, including that of driving ‘economic growth by making England the default location for world-class health services research’.

Applications to use the data are to be reviewed by the HSCIC's Data Access Advisory Group. If requests to use data are classified as sensitive and identifiable, researchers are subject to additional requirements, including that of undergoing scrutiny from the Confidentiality Advisory Group of the Health Research Authority. This group replaced the PIAG and an interim body (the Ethics and Confidentiality Committee operating under the National Information Governance Board). It continues the role of advising on data use for research when individual consent has not been obtained on the basis of so-called Section 251 approvals.

Other changes relevant to the creation of *care.data* included amendments in 2012 to the NHS Constitution^21^ to offer a ‘pledge’ to inform English NHS patients about research studies in which they might be eligible to participate, and also, crucially, an expectation that patients would be willing to share their medical information for healthcare planning and for research purposes. The relevant section—‘Respect, consent and confidentiality’ (p. 8)—explains that patients have the right to request that their information should not be shared but also that such requests may not be upheld if the public interest is found to outweigh the individual case.

Thus was the administrative infrastructure for *care.data* created—but not, it seems, the social licence. The amendments to the Health and Social Care Act 2012 and to the NHS Constitution seem to have been based on a re-imagining and re-responsibilisation of patients as active citizens. There has been an assumption that the implicit social contract underpinning the NHS would mean most patients would be happy to support sharing of their personal data in the interests of the six aims of the *care.data* programme, including economic goals to be realised through research.

Yet the extent to which confidence in such assumptions was well-founded was not clear. Previous social science research suggests a nuanced and delicate understanding of societal support for, and cooperation with, health research is needed.^7^ It has found that the public's support and tolerance for research, and its associated risks, often depends far more on an often fragile set of cues about the safety and social good of research participation, and on institutional and professional credentials, than it does on the formal architecture of research regulation, or on rational assessment of the detail of information sheets or other documents aimed at gaining ‘informed consent’.^22^ ^23^

Further, most of what is known about patients’ support for research is based on quite particular examples of research participation—often those where patients already have an interest in a medical condition and where they are asked for quite specific consent to a project or programme. Where patients specifically consent to a particular study or programme of research, they may (depending on the study) expect the possibility of some personal benefit, as well as being able to experience secondary benefits of their consent, such as the ‘warm glow’ associated with altruistic behaviour that benefits an identifiable community (eg, of those with a specific disease).^24^ The extent to which the findings of this body of research about participation in specific, relatively well-bounded studies or cohorts by defined, consenting patients can be generalised to the broader conception of NHS citizenship implicit in the new policy direction is not clear.

What is clear is that individuals' cooperation with specific research studies is usually secured through three principal mechanisms: their expectations about how research is conducted and regulated; their trust in the institutions and individuals who recruit them; and their beliefs in the wholesomeness and public value of the research endeavour. More broadly, the public legitimacy and acceptability of health research rests heavily on its status as a socially valuable enterprise conducted in the service of the public good.^25^

There are many reasons to doubt that *care.data* could *reasonably* assume that the public would automatically confer upon it the same legitimacy and endorsement as that enjoyed by research where individual informed consent is sought and clear information about study aims is provided. For instance, the mobility of electronic data and the practical difficulties of specifying in advance the research questions for which data might be used or the populations to be studied mean that *care.data* was in many ways quite distinct from conventional research projects. Three threats to the social licence faced by *care.data* are especially important in explaining the challenges it faced: (i) defects in the warrants of trust provided for *care.data*, (ii) the implied rupture in the traditional role, expectations and duties of general practitioners (GPs), and (iii) uncertainty about the status of *care.data* as a public good.

## Warrants of trust

Ensuring that people are aware of how data from medical records has been or might be used, and the protections that are in place, might be assumed to be critical to ensuring their confidence that such use is legitimate and well-governed.^10^ A systematic review of public awareness of, and views on consent to, the secondary use of medical records for health has identified a generalised lack of awareness and understanding.^26^ Yet level of ‘awareness’ itself may not be a reliable guide to people's faith in a system. Individuals’ willingness to cooperate with health research often depends on powerful heuristics rather than detailed understanding of research governance procedures and standards.^7^ Even when they agree to take part in specific studies, it is not the detail of consent forms and information sheets that matters to participants so much as an overall faith in the legitimacy of the endeavour and a reassurance of protection from risk.^22^ ^23^ ^27^ ^28^ Important, too, is a belief that agreeing to participate in research will not make individuals vulnerable to risk of harm, exploitation or charges of gullibility.^27^ This is not to imply that consent forms and information sheets are of no value or relevance; on the contrary, they act as ‘symbolic tokens’,^23^ as vitally important warrants of the trustworthiness of the process.^22^

Thus, a lack of detailed awareness might have mattered little in the roll-out of *care.data* had the right warrants of trust been in place. But the approach adopted did not appear to provide them. The implied consent model underlying *care.data* did allow for patients to opt out, but the process for ensuring that they were enabled to do so was precarious.^29^ The practical delivery of the requirement to ensure patients were aware of *care.data* and their right to opt out proved problematic. The NHS England information leaflet about the initiative was addressed to households rather than individuals and might have appeared to be unsolicited junk mail.

For those seeking to improve their understanding, the leaflet itself did not appear to be fully informative. Entitled ‘Better Information Means Better Care’, it made no explicit reference to *care.data* other than to direct those seeking further information to an NHS website. It did not include an opt-out form. The benefits of information sharing were explained in the patient information leaflet, but the risks were not.^30^ HSCIC's own acknowledgement that ‘a small risk of re-identification due to small numbers/rare diseases’^31^ was not conveyed. Questions about who would access data, for which purposes and on which terms, were also left unclear, despite evidence showing that the public wanted more information about how governments and companies collect, share and use data.^32^ Additionally, it was not clear that busy GPs would have the time or sufficient information to engage in complex discussions with patients wishing to opt out, nor was any extra resource or support provided for the task, even for vulnerable groups such as care home residents. The concerns of GPs regarding losing control of patient data during this process were also not addressed. Further, few opportunities were available to the public to influence the direction of policy.^33–35^

Overall, in contrast to the cues usually available to research candidates, the warrants of trust provided by *care.data* appeared inadequate and were likely to have undermined the social licence rather than strengthen it. Lack of detail in the information sheets was unlikely to have been the direct cause of the failure to secure the social licence, but it does provide visible evidence of how the operationalisation of the programme was founded in poor understanding of what was needed for the public and professionals to have trust and confidence in it. Inadequate consultation and engagement meant a failure to take into account ‘the right things’ and thus secure the social licence.

## Rupture in traditional expectations

The legitimacy of health research draws heavily and crucially on people's trust in organisational and professional credentials,^7^ including the trust placed in professionals who have a duty of confidentiality^36^ and relationships developed through continuity of care and empathy.^37^ GPs are constrained both by the common law duty of confidentiality and statutory requirements under the Data Protection Act 1998 and Human Rights Act 1998 to respect patient confidences and to process personal data or disclose confidential information only within clearly defined parameters. However, in neither legal regime is the protection of privacy absolute. A 2013 review of the system of information governance in health and social care services clarified, for example, that legally patients do not own their data.^9^ Notwithstanding, what is legally permissible and what is socially acceptable do not necessarily coincide. For some, *care.data* represents a significant and unwelcome alteration to traditional understandings of the private and confidential nature of the relationship between GP and patient.

Some accounts argue that routine monthly extraction of electronic individual level patient data from GP records represents a step-change in terms of the level of intrusion into patients’ private lives, to the extent that it has been suggested that it erodes the fundamental right to respect for private life offered by the Human Rights Act. Concerns have been expressed that patients may not wish intimate details of their medical records to be accessible to those outside of their ‘circle of care’,^38^ and might decline to trust and confide in their healthcare providers if they know that their information will be shared.^39^ The law has long recognised the importance of protecting this trust for both the private and public interests that it serves (see, for example, the cases of X v Y (1988) 2 ALL ER 648 and also Campbell v Mirror Group Newspapers (2004) UKHL 22). These concerns are linked in part to the advances in technological capability associated with ‘big data’^32^ which offer the opportunity to address novel research questions^40^ but also generates new ethical dilemmas in relation to re-identification of individuals^14^ ^41–43^ or losses of data and other intrusions associated with risks to anonymity and privacy. Again, the extent to which the social licence for these potentially profound alterations to the nature of the doctor–patient relationship was secured for *care.data* is in question.

## Status of *care.data* as a public good

Values of reciprocity and fairness underpin the legitimacy of health research: if people participate voluntarily, they expect that their contribution will be used to improve the care of others, and that their good faith will not be exploited.^23^ ^24^ Much depends, therefore, on the extent to which uses of personal data are seen as serving the public interest and conducted by those with a public interest orientation. Yet neither of these features of the social licence seems to have been fully addressed for *care.data*.

The ‘public regime of justification’^36^ that was provided for *care.data* stressed its benefits at a national rather than an individual level. Of the six aims of *care.data* (box 1), the first five are rooted in the use of data to improve quality and delivery of care and the governance of healthcare, with the recommendations of Sir Robert Francis arising from his damning report on Mid Staffordshire NHS Trust^44^ invoked to supply part of the motivation. However, there are indications that members of the public expect to see benefits of such data use made explicit.^26^ The sixth aim of *care.data* refers to research, but in a way that links it explicitly to economic growth.

The multiplicity of aims towards which *care.data* is directed, and the linking of *care.data* and research to the so-called ‘health and wealth’ agenda, may be an unfamiliar rationale for citizens used to a public service model of healthcare.^45^ Patients may mistrust commercial interests, especially where these might be perceived as profiteering or resulting in excessive profit^26^ ^32^ ^35^ ^46–48^ or where patients have concerns about the extent to which risks and benefits are evenly distributed and whether their contributions to research will be reciprocated by a contribution to the public good. The persistent problem of non-publication of study results is just one element of how non-reciprocation may manifest.^49^ Other concerns may be linked to questions of who will be able to access *care.data* for which purposes with which risks, how the credentials of bona fide researchers can be established, and what mandate commercial organisations will have to use data that originated from private consultations between patients and their GPs.

The complexity is amplified by the institutional fragmentation of the NHS in England, which is no longer a single unitary public sector entity. Transfer of information ‘within the NHS’ in practice means transfer between entities with distinct legal status. This takes place in a complex framework of statutory provisions and legal contracts, and it increasingly occurs with those who are private contractors of services to the NHS or may operate outside the NHS entirely. Concerns about insurance companies and others having access to personal data^5^ suggest that considerable further dialogue between the public, affected stakeholders such as GPs, and policy makers is required before a social licence can be said to be in place for *care.data*.

## Conclusions

The concept of a social licence offers insight into why the implementation of *care.data*, an initiative to extract data from routine general practice consultations for use in research and other activities, has met with concern and controversy. Although the infrastructure was in place, the activities were perfectly lawful, and a case had been made for the possible benefits that might be generated, the experience of *care.data* starkly exposes an enduring truism about the limits of law: legal authority does not necessarily command social legitimacy. A parliamentary majority may allow legislation to be passed, but that does not equate to a societal seal of approval or to securing the trust and confidence of patients, citizens, healthcare professionals and researchers. Securing a social licence may require something other than a legal mandate.

Although England has remained firmly on the legislative path, different solutions to the same challenges relating to the use of personal medical information have been found north of the border.^50^ Rather than legislative change, actors operating within the existing legal frameworks have worked together under the Scottish Health Informatics Programme (SHIP) to develop mechanisms of principled proportionate governance—building on public engagement exercises—to deliver responsive, risk-based approaches to data linkage for health research. Consent, anonymisation *and* authorisation are all available as governance tools to be deployed, as appropriate, in any given data linkage proposal after an assessment of the risks and tolerances involved.^51^

As the Scottish example shows,^51^ this does not mean that unanimous social consensus is required for all developments. Rather, a social licence for research will require, as a minimum, that certain conditions of social engagement have been respected. Genuine dialogic engagement that might result in a broad licence must be distinguished from more narrowly focused public relations exercises that seek to ‘capture’ the public, that is, to persuade the public of the legitimacy of decisions already taken by experts,^33^ ^35^ and from simple ‘awareness-raising’ information exercises. While legitimate disagreement is inevitable, if a social licence is to be maintained, both the final result and process used to achieve that result must be one which reasonable citizens can at least recognise as defensible on the grounds that it reflects common social values and goals.

Trust and confidence in research governance or the ‘social licence for research’ depends upon ideas about the public good that are not straightforwardly synonymous with the aim of increasing the UK's gross domestic product. What patients care about as patients cannot be equated with what patients care about as citizens who are part of a much wider social endeavour. If *care.data* is to succeed, patients need to have the confidence that their medical records will be held securely, anonymised appropriately, and that secondary use of this personal data is in the public interest: the conditions of the social licence need to be respected in ways that go beyond compliance laid down in a legal framework. Necessary—and hopefully sufficient—conditions for social licence include: (i) *reciprocity*, which must begin with sound two-way communication, (ii) *non-exploitation*, which must exclude the spectre of disempowerment, and (iii) *service of the public good*, which need not exclude a wealth agenda so long as there is confidence that research governance and information governance systems can hold researchers, and others with custodial responsibility for medical information, to account.
